# National Veterinary College

**Published:** 1895-09

**Authors:** 


### NATIONAL VETERINARY COLLEGE,

The fourth annual announcement has just been received and the following changes are noted in the faculty: George Jobson, V.S., house surgeon, professor of anatomy and theory of shoeing, has been succeeded by Wm. P. Fink, D.V.S., graduate of the Class of 1895, as professor of anatomy, the theory of shoeing having apparently been discontinued. Prof. Dawson has added to his chair the subject of bacteriology, which was formerly taught by Dr. Theobald Smith as a special lecturer. Chas. R. Clarke, M.D., has been added to the corps of instructors as professor of histology. C. F. Hadfield, D.V.S., graduate of the Class of 1895, has been added, under the head of special lecturers, as demonstrator of anatomy. The subject of canine practice appears to have been dropped from the subjects taught.

The course commences on October 3, and the final examination commences April 6. We note no change in the marticulation examination. The didactic lectures commence at 5.30 P. M. each day and end at 9.30 P. M. The clinical lectures and demonstrations from 9 A. M. to 12 noon.

The following has been eliminated from the requirements for graduation: Candidates who fail to pass in three branches or less will be permitted to present themselves for an examination in those branches during the Christmas holidays following. If successful in this midwinter-term examination their diplomas will be granted at that time.

Under the head of a three-year graded course recommended by the faculty, the important statement is made that, while this course is now an optional one, after the coming session it will be made obligatory, in order to make eligible those students who wish to practise in States having laws regulating practice. No fees are charged for the optional third-year course.

A post-graduate course will be established in connection with the coming session, where the subjects of contagious diseases, meat inspection, sanitary police, special therapeutics, bacteriology, immunization, general and special pathology, histology, comparative osteology and myology, chemistry and toxicology, zoötechnics, and special economic botany. These lectures are designed for veterinary graduates, and a certificate of attendance will be granted. The fee for this course has been placed at $75.

---

